# Common Attentional Constraints in Visual Foraging

**DOI:** 10.1371/journal.pone.0100752

**Published:** 2014-06-25

**Authors:** Árni Kristjánsson, Ómar I. Jóhannesson, Ian M. Thornton

**Affiliations:** 1 Faculty of Psychology, School of Health Sciences, University of Iceland, Reykjavik, Iceland; 2 Department of Cognitive Science, Faculty of Media and Knowledge Sciences, University of Malta, Msida, Malta; University of Sussex, United Kingdom

## Abstract

Predators are known to select food of the same type in non-random sequences or “runs” that are longer than would be expected by chance. If prey are conspicuous, predators will switch between available sources, interleaving runs of different prey types. However, when prey are cryptic, predators tend to focus on one food type at a time, effectively ignoring equally available sources. This latter finding is regarded as a key indicator that animal foraging is strongly constrained by attention. It is unknown whether human foraging is equally constrained. Here, using a novel iPad task, we demonstrate for the first time that it is. Participants were required to locate and touch 40 targets from 2 different categories embedded within a dense field of distractors. When individual target items “popped-out” search was organized into multiple runs, with frequent switching between target categories. In contrast, as soon as focused attention was required to identify individual targets, participants typically exhausted one entire category before beginning to search for the other. This commonality in animal and human foraging is compelling given the additional cognitive tools available to humans, and suggests that attention constrains search behavior in a similar way across a broad range of species.

## Introduction

Several lines of evidence suggests that predators often select prey of the same type in non-random sequences or “runs” that are longer than would be expected by chance [1]–[3], for review see [4]–[5]. Such foraging is thought to be mediated by internal templates or ‘search images’ that bias the way the environment is sampled [1], [6]–[7]. When food items are conspicuous, a predator may switch between available sources at random, interleaving short runs of different prey types. However, when prey are hard to detect, or cryptic, for example due to camouflage, a predator may focus on a single food type, effectively ignoring equally available sources [6], [8].

Although originally based on direct observation in the wild [1], experimental studies of foraging by ‘search image’ have typically inferred run-like behavior from patterns of free-choice [3], [9]–[11] or serial-detection responses [12]–[15] rather than directly measuring the sequence in which items are taken [4], for exceptions, see [1], [16]. Here we introduce a simple iPad task to directly measure “foraging” sequences in humans when confronted with multiple targets from different categories.

Traditionally, studies of human search have involved a single target that must be located amongst a variable set-size of uniform distractors [17]–[20]. A growing realization that real-life search behavior can often be more complex has prompted increased interest in multiple-target search, much of it directly inspired by animal foraging studies [21]–[27]. Our new approach allows simultaneous exploration of important phenomena from both the animal foraging and the human search traditions using a single task. Our overall aim was to gain a better understanding of how humans coordinate search when multiple targets from different categories must be located.

**Figure 1:**
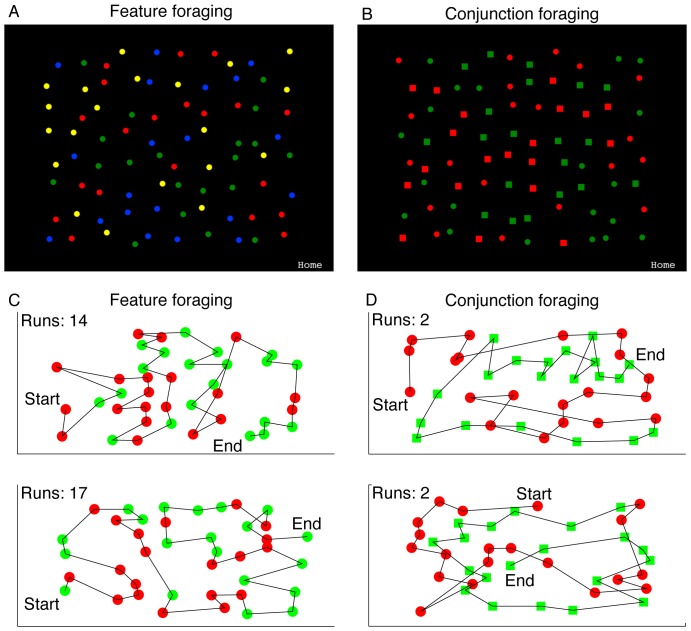
Example trials and foraging paths. Panel A shows the feature foraging condition, where the task is to cancel all red and green circles while ignoring blue and yellow (or vice versa). Panel B, shows the conjunction foraging condition where the task is to cancel out all the red squares and the green circles (or vice versa). Panels C and D show typical foraging paths for the feature and conjunction conditions respectively. To explore the search space in these two conditions we suggest the reader locate the “Start” symbol in each example and follow through the sequence of symbols.

Figure 1 shows our displays that consisted of many coloured items randomly distributed on an iPad screen. On each trial, participants searched for and tapped on all targets as quickly as possible. Each participant “foraged” in this way for two target categories and ignored two distractor categories. In the “feature” condition, targets and distractors were distinguishable by a single dimension: color. For example, targets might be the 20 red and 20 green disks and distractors, the 20 blue and 20 yellow disks (Figure 1A). Once a target was tapped, it disappeared. If a distractor was tapped, the trial immediately finished and an error message was displayed. Participants were told to respond as quickly as possible, while avoiding errors.

In the “conjunction” condition, both color and shape were used to define the categories. For example, targets might be the red circles and green squares and distractors the red squares and green circles (Figure 1B). From the human visual search literature, we know that target complexity – the number of features that define a target, rather than visibility *per se* – is a prime determinant of search efficiency [28]. We therefore used the conjunction manipulation to increase attentional load, rather than varying the visibility of targets, as is typically done with cryptic prey in animal studies [6].

How do humans forage when presented with multiple targets from more than one category? Will search behavior be characterized by long “runs” in which targets of a single type are sequentially selected, analogous to cryptic prey selection in other species and as predicted by findings on attentional priming [29]–[30]? Or will foraging be dominated by the local layout of the display leading to frequent alternations between target categories? Most importantly, how will difficulty (i.e. feature versus conjunction foraging) affect the pattern of runs?

## Methods

### Participants

Sixteen students at the University of Iceland, aged from 22 to 39 years (9 females; M = 28.3 years, SD  = 4.6 years) participated in this study. All reported normal or corrected to normal vision, were right handed and gave written, informed consent. All aspects of the experiment were reviewed and approved by the departmental ethics committee at the University of Iceland and thus conformed to the ethical guidelines set out by the Declaration of Helsinki for testing human participants.

### Equipment

The stimuli were displayed on an iPad 2 with a screen dimension of 20×15 cm and an effective resolution of 1024×768 pixels. The iPad was placed on a table in front of the participant in landscape mode, so that viewing distance was approximately 50 cm. As viewing distance could not be precisely controlled, we report distance measures in both pixels and degrees visual angle. Stimulus presentation and response collection were carried out by a custom iPad application written in objective-C using Xcode and Cocos2d libraries.

### Stimuli

In the feature-based foraging task, the targets were red and green disks and the distractors were yellow and blue disks for half of the participants while for the other half this was reversed. In the conjunction foraging-task, the targets were red squares and green disks and the distractors were green squares and red disks for half of the participants (again reversed for the other half). There were 20 stimuli in each group, drawn on a black background (see Figure 1). The diameter of targets and distractors was 20 pixels, approximately 0.46° visual angle.

The items were randomly distributed across a non-visible 10×8 grid that was offset from the edge of the screen by 150×100 pixels. The whole viewing area therefore occupied 15×12 cm (approximately 17.1×13.7°). The exact position of individual items within the grid was jittered by adding a random horizontal and vertical offset to create a less uniform appearance. Gaps between rows and columns ensured that items never approached or occluded each other. The overall spatial layout and the location of targets and distractors was generated independently on every trial.

### Procedure

The experiments were run in a quiet room with normal illumination. The participants' task was to tap all targets as quickly as possible using the index finger of their right hand. The targets disappeared immediately following the tap. If participants tapped one of the distractors the trial ended, an error message was given, and a new trial started. Each participant participated in 25 trials of each task (in counterbalanced order). One trial refers to a completed sequence where all 40 targets were cancelled. The first 5 trials were considered training and were excluded from analyses, leaving 20 test trials for each condition.

### Data Analysis

Raw data from all 16 participants can be found in File S1 of the supplementary material. The performance of one participant (P13) was consistently >3.0 SD from the mean of the group on two of our main dependent measures, response time and distance moved. This participant also had a unique profile of run-like behavior. For the sake of completeness, data from this participant are included in the tables and graphs showing individual performance, and are discussed in the relevant sections, but were excluded from statistical analysis. Overall, error rates were typically very low (<2%), and our analysis focused on performance within the 20 correct trials of remaining 15 participants.

**Figure 2:**
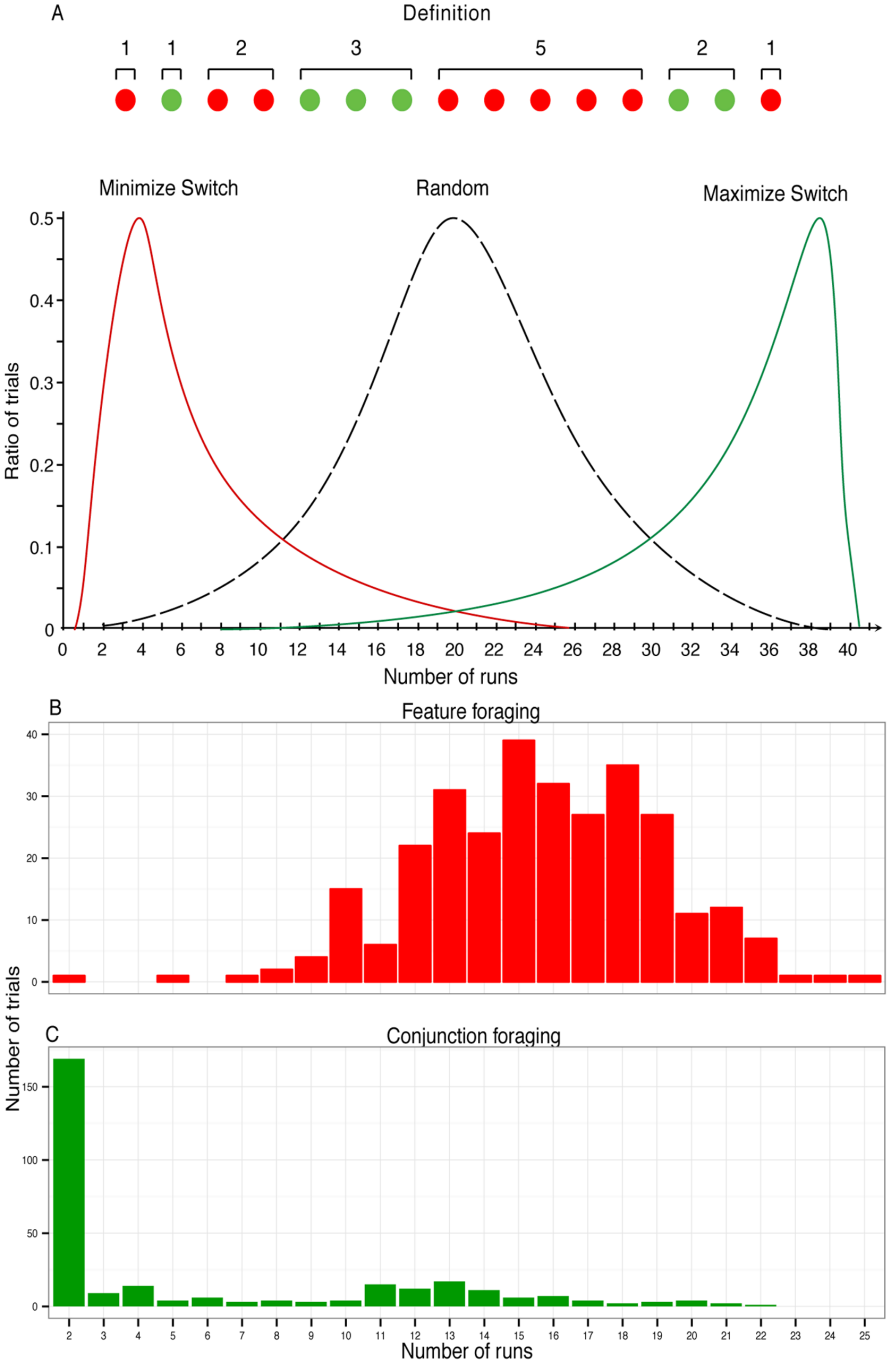
Hypothetical and actual distributions for the number of runs. Panel A shows a sequence containing 7 runs and sketches three hypothetical distributions for overall foraging patterns. Note that these hypothetical distributions are simply caricatures meant to illustrate extreme strategies. If foraging were random we would expect a normal distribution of runs as the middle curve reflects. If participants shift repeatedly between targets during foraging the distribution to the right would be observed but if switching is minimized the distribution on the left would be observed. Panels C and D show the actual distributions obtained in the feature and conjunction foraging conditions, respectively, collapsed across participants. Note the change in the X-axis relative to Panel A and the difference in the Y-axis between the feature and conjunction panels. The histograms clearly show that the runs are much fewer (hence longer) in conjunction than in feature foraging.

In line with previous studies [2], [3], the number of “runs” on a given trial was our primary dependent measure. For statistical purposes, a run may be defined as “a succession of one or more types of symbols which are followed and preceded by a different symbol or no symbol at all” [31]. Here, our symbols are the target items, either defined by a single color feature or by the conjunction of color and form. With our displays, the maximum number of runs per trial is 40, if a switch occurs after every response. The minimum run-number is 2, if all targets of one category are cancelled before the other. Selecting by chance would yield an average of 20 runs. The concept of a “run” and theoretical foraging distributions are illustrated in Figure 2A. Examples of actual search paths through the displays are shown in Figure 1C–D.

To directly compare the number of runs across conditions, and to explore whether performance changed as a function of time, we used a 2 (Condition: Feature/Conjunction) ×20 (Trial) repeated measures Analysis of Variance. This same model was also applied to examine cumulative response time and distance measures. In this initial study, we report and analyzed the total time taken to make all 40 responses and thus complete a trial, referred to below simply as ‘response time’. Similarly, our distance measure was the sum of the Euclidean distance between successive targets, measured in pixels, which we refer to simply as ‘distance travelled’.

Given the large number of levels in our Trial factor, we adopted a conservative approach with regards to possible violations of Sphericity. That is, we applied Greenhouse-Geisser corrections to all cases where Epsilon values were less than 0.75. This resulted in corrections being applied to all main effects and interactions involving Trial.

To determine if run behavior was random, we used the One Sample Runs Tests to examine the data of each participant separately. Specifically, we asked if each individual trial appeared random, conducting 20 separate analyses per participant and using Bonferroni correction to adjust the level of alpha for multiple tests. We thus quantified the proportion of trials of that were non-random at the p<0.05 level for each participant and compared these across conditions using a paired t-test.

Finally, to explore the relationship between dependent measures, we ran a series of simple correlations to examine whether the number of runs appeared to predict overall response time and distance travelled.

## Results

Histograms of the run behavior of 15 participants are shown in Figure 2B and C. When target selection was easy (feature condition, Figure 2B), participants alternated frequently between target categories, leading to a relatively high number of runs (Mode  = 15). Runs test analyses indicated that on average only approximately 3 out of 20 trials per participant in this condition were classified as non-random. Table 1 shows a summary of the number of trials that contained non-random runs for each participant. Individual differences may even slightly inflate this estimate of non-random behavior for the feature condition and several participants always switched randomly.

**Table 1:** Number of trials classified as non-random as a function of condition and participant.

Participant Number	Feature Condition	Conjunction Condition
1	1	19
2	0	19
3	0	20
4	1	2
5	0	20
6	0	20
7	4	17
8	0	4
9	0	14
10	6	19
11	1	0
12	0	19
13	19	20
14	5	9
15	1	20
16	6	20
**MEAN**	**2.8**	**15.1**
**SD**	**4.9**	**7.2**

The foraging pattern was quite different in the conjunction condition (Figure 2C, note the change of scale). Now, the majority of trials consisted of just two long runs where one target category was exhaustively cancelled before the other was selected (Mode  = 2). On average, approximately 15 out of 20 trials for each participant were now classified as non-random, again with some consistent individual differences (see Table 1). Not surprisingly, a direct comparison of the number of non-random trials between the two conditions indicated a significant difference, t(14) = 6.2, p<0.001.

To test for changes in run behavior over time, we conducted a 2 (Condition) ×20 (trial) repeated measures ANOVA on the number of runs. There was a main effect of Condition, F(1,14) = 58.3, MSE  = 239.3, p<0.001, 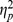  = 0.8, but no significant main effect of Trial and no interaction (see Table 2 for details). An identical pattern was seen in the analysis of distance travelled and response time. There was consistently more movement across the screen in the conjunction condition (M = 6044 px) than the feature condition (M = 4842 px), reflecting extended run-like behavior, F(1,14) = 115.6, MSE  = 1874496.8, p<0.001, 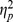  = 0.9, but no effect of Trial and no interaction (see Table 2). Response times were also longer in the conjunction (M = 14.1 sec), than in the feature (M = 11.9 sec) condition, reflecting the cost of target complexity, F(1,14) = 68.9, MSE  = 22146752.8, p<0.001, 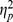  = 0.8. Again, there was no effect of Trial and no interaction (see Table 2).

**Table 2:** ANOVA summary table for three dependent measures: Number of runs, movement across the screen and trial completion time.

Dependent Measure	Factor	df	Mean Square	F	Sig	Partial Eta Squared
Runs	Condition	1	13958.78	58.33	.00	.81
	Error(Cond)	14	239.33			
	Trial	6.87	18.04	.78	.60	.05
	Error(Trial)	96.12	23.15			
	Cond x Trial	6.93	20.37	.86	.55	.06
	Error(Int)	97.02	24.11			
Movement	Cond	1	216719076	115.62	.00	.89
	Error(Cond)	14	1874496.82			
	Trial	7.01	496340.32	.79	.60	.05
	Error(Trial)	98.16	628612.61			
	Cond x Trial	8.88	421652.51	.88	.54	.06
	Error(Int)	124.28	477806.79			
Response Time	Condition	1	1525943812	68.90	.00	.83
	Error(Cond)	14	22146752.85			
	Trial	8	6318230.31	1.637	.12	.11
	Error(Trial)	112.04	3858811.52			
	Cond x Trial	6.78	4969419.10	.92	.49	.06
	Error(Int)	94.87	5385173.90			

**Figure 3:**
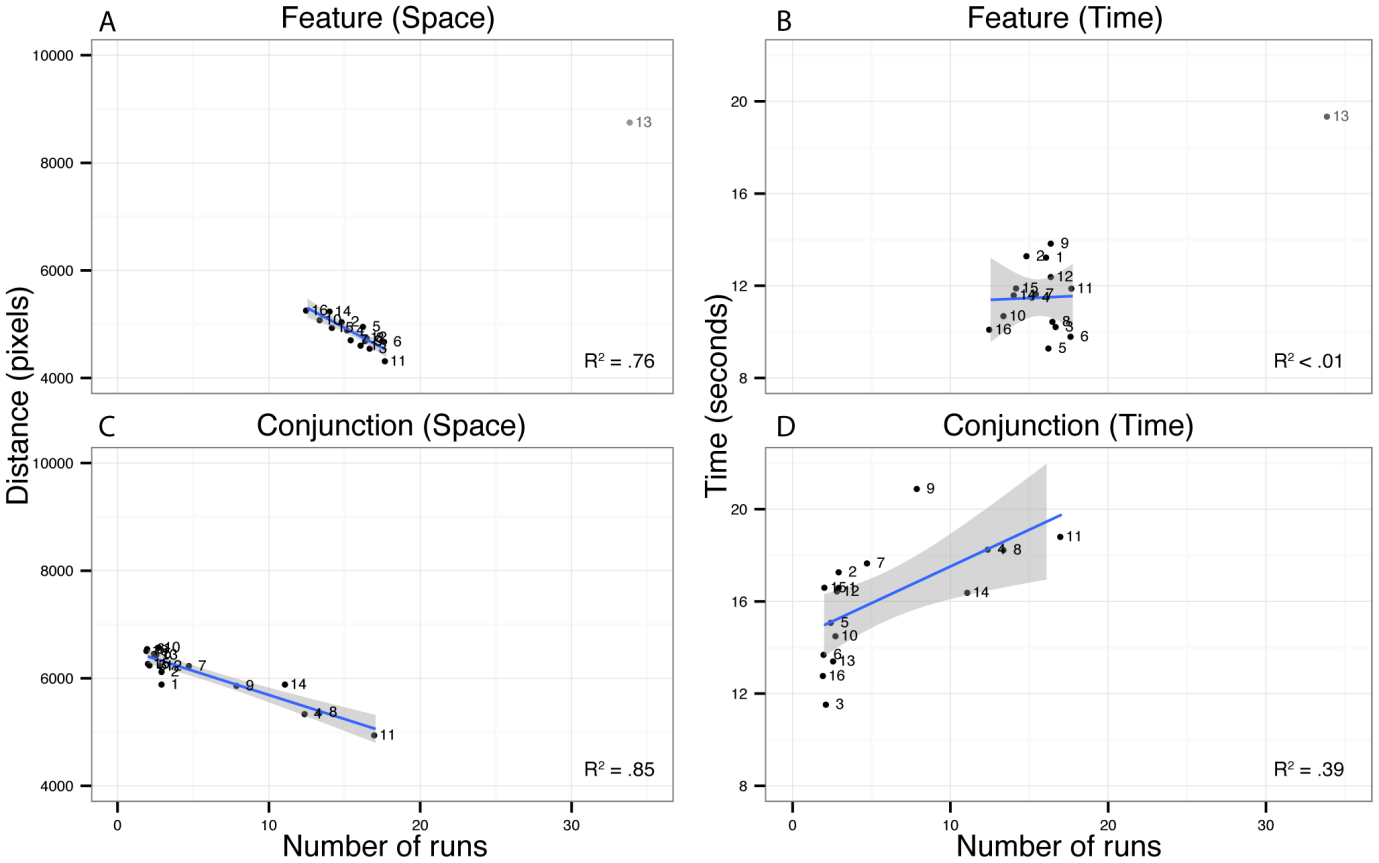
Distance between consecutive taps and mean trial finishing time as a function of run length. Panels A and B (feature foraging) and C and D (conjunction foraging) show the relationship between mean number of runs and (LEFT) distance travelled and (RIGHT) mean trial finishing time for each participant. The shaded areas represent 95% CI of the fitted lines.

Figure 3 shows the relationship between our main dependent variable, the number of runs, and the distance and time measures. The number of runs clearly has a direct impact on the distance travelled in both feature and conjunctions, and hence the energy expended on a given trial. That is, on a participant by participant basis, higher numbers of runs leads to less overall movement across the screen. Figure 3 (A–B) plots the mean distance moved per trial as a function of number of runs for all participants in the two conditions. In the feature condition, with the exception of one obvious outlier (P13), there is a very consistent negative correlation between distance travelled and the number of runs, r = −0.87, p<.001. The pattern in the conjunction condition is very similar, r = −0.92, p<.001, although this relationship appears to be strongly influenced by a subset of participants. We return to the behavior of these participants shortly and further examine the data of P13 in the Discussion.

Figure 3 (C–D) plots the mean response time per trial as a function of number of runs for all participants in the two conditions. There is clearly little relationship in the feature condition (Figure 3C) between the number of runs and trial completion time, r = 0.08, p = 0.78. In the conjunction condition (Figure 3D), while there is an overall positive relationship, r = 0.56, p = 0.03, this pattern is again clearly influenced by the same subset of participants who had the unusual distance behavior.

**Figure 4:**
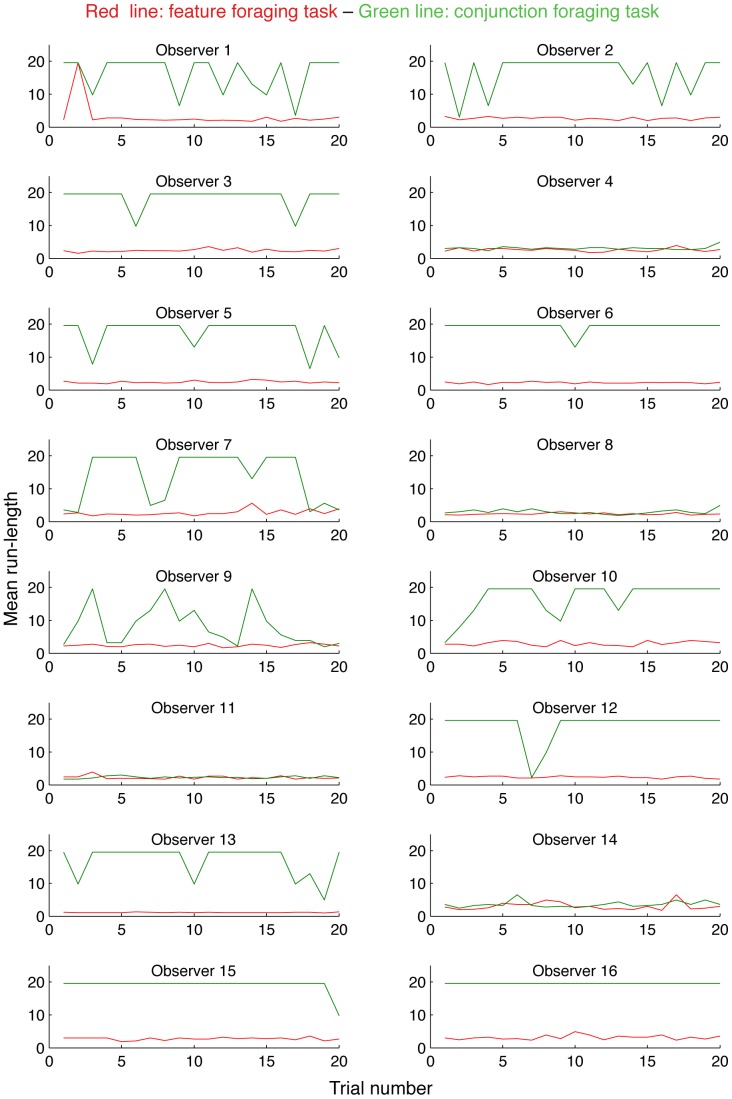
Average run length as a function of trial for each participant in both feature and conjunction foraging conditions. For most participants, the difference in run length (and therefore number of runs) between feature and conjunction search is clear. Note however, that participants 4, 8, 11 and 14 have essentially identical feature and conjunction patterns.

Figure 4 provides another way to visualize the results, also allowing exploration of individual participants. Each panel shows data from one participant, and the red and green lines plot the *average run length* per trial for the feature and conjunction conditions respectively. Run length has a simple inverse relationship to the number of runs, such that trials consisting of few runs will have longer sequences and vice versa. It is immediately obvious that four participants (P4, P8, P11 & P14) have essentially identical feature and conjunction foraging patterns. That is, for these participants, in contrast with others, the increase in target complexity does not influence the pattern of runs and they switch between targets at a similar rate for both conditions. This strategy appears to have both costs and benefits, as they move less across the screen, but take slightly longer to complete each trial (Figure 3C–D).

There are several other things to note from Figure 4. First, behavior does not appear to change systematically as a function of trial number. There is no adaptation or consistent change of strategy for any participant as conditions progress. Second, variability in the feature condition is very small, both within and across participants. Only one participant (P1) ever utilized long runs with a single target category, and only on a single trial. Third, in the conjunction condition, in addition to the four participants already mentioned, the foraging is more variable, with several participants alternating between long and short runs. With the exception of P9, this alternation to shorter runs tends to be brief and isolated, suggesting, that such behavior is quite effortful during conjunction foraging. Consistent with this idea, P9 has the longest trial completion time of any participant.

## Discussion

We believe our new foraging task has great potential for exploring search-related phenomena beyond the tasks typically examined with human participants. The task can be administered quickly and easily and can be modified in a variety of ways. In this initial study, using two target and two distractor categories, we have already identified a number of interesting findings that can form the basis of further investigation.

First, we have demonstrated that humans can easily switch between search categories if individual target items are easy to detect and effectively “pop out”. Second, we have shown that for the majority of participants, increasing target complexity completely alters foraging behavior, minimizing switches in favor of exhaustive, single-category searches. Finally, we identified a subset of individuals whose foraging ability was apparently immune to the influence of target complexity. We discuss each of these findings in turn.

Our first finding would appear to parallel the behavior of animals searching for conspicuous prey. Both standard [32]–[33] and attention-related prey models [8] predict that when all prey are easy to detect, predators should feed equally on all available sources [5]–[6]. In line with these predictions, a number of studies using free-response and/or serial detection designs have failed to find feeding patterns consistent with the use of runs when prey are conspicuous ([3], [10]–[11], [34]–[35]. In the current easy condition, where single features distinguish targets from distractors, it appears that participants can easily hold two templates or search images in working memory simultaneously and alternate randomly between them. The precise sequence of responses would thus appear to be mostly determined in a bottom-up manner by the overall layout of the display rather than by top-down attentional constraints.

Our second finding conforms exactly to a key prediction of the “attentive prey model” [8], the prediction that when attentional load is high, an animal “should search only for items of a single prey type and ignore all other types” [6]. Specifically, in our conjunction condition, twelve out of 16 participants went from switching easily between two target types to focusing solely on one target type, finishing it, before switching to the other. Under these conditions, the “environment” appeared to have little influence on search behavior and the sequence of responses was apparently determined by the currently active search image.

To our knowledge, this is the first direct demonstration of exhaustive, run-based foraging behavior in humans. Finding such a pattern suggests that attention may influence behavior in a very similar way across a broad range of species. It would also seem to validate the use of feature/conjunction manipulations – in addition to target visibility – as a way of exploring the role of attention during foraging, at least for humans.

As noted in the Introduction, only two previous studies have explicitly measured the sequence of responses to multiple targets in the context of foraging [2], [16]. Neither of these studies contained a manipulation where behavior was switched between random and run-based search. Dawkins [2] studied the foraging pattern of chicks feeding on rice, dyed either orange or green, on an orange background. Each feeding tray thus contained both conspicuous and cryptic items. Non-random feeding sequences were observed on all trials, but whether runs differed between the conspicuous and cryptic grains of rice was not reported. Clearly, the interleaving of the two types of food on the same trial makes direct comparison with the current work problematic.

Bond [16], in a very elegant and innovative study, had human participants sort entire sets of physical wooden beads into color categories, dropping them from a sorting tray into target tubes where micro-switches recorded response times. Sorting took place on a mat with a uniform color and all beads were thus effectively conspicuous. Difficulty was manipulated by varying the similarity of the bead colors. Non-random sequences of runs were observed with both easy and hard versions of the task, and the number of runs did not differ as a function of difficulty. The appearance of runs with what were effectively ‘conspicuous’ items, suggests that other aspects of the task – the need to select and sort between four categories – may have increased the demands sufficiently to limit attention, relative to our simple search and cancellation task, an issue we return to below.

The current work not only adds a third example of directly measured run-like performance to the literature, but possibly more importantly, provides a very effective means to switch behavior between random and run-based foraging. Indeed, for the 12 participants we have considered so far in this discussion, the effect of the conjunction manipulation was quite dramatic. Examination of Figure 4 suggests that there were very few trials in which run patterns other than two very long sequences were obtained. We speculate that this reflects an inability to maintain two conjunction templates at the same time, exceeding available attentional resources. While we cannot rule out that the use of such long runs simply reflects a strategic decision, such an explanation ignores the impact of attention – why were there no examples of such a strategy in the feature condition? – and provides no account for the limited within- and between-participant variation.

Clearly, an important avenue for future research will be to see if we can manipulate task difficulty in a more graded fashion, to produce patterns of behavior that fall somewhere in between random, and exhaustive search. Fortunately, the current task is extremely easy to adapt for this purpose. Difficulty could, for example, be varied by making one target category feature-based and the other conjunction-based, following the mixed design of Dawkins [2]. We could also easily vary the similarity between target categories and/or increase their number, following Bond [16]. Finally, although we have focused in this initial paper on the feature/conjunction manipulation, the task would also seem ideal for directly exploring the impact of target visibility. That is, the simple geometric shapes used here could be replaced with virtual [35] or photographic [36], [37] images of prey to directly explore crypsis by parametrically varying the background.

So far in this Discussion, we have focused on the pattern of feature/conjunction foraging displayed by the majority of our participants. Our third main finding, however, points to an important additional factor that needs to be considered in studying human foraging. Specifically, 4 participants showed no variation between feature and conjunction search, apparently violating the predictions of the attentive prey model. We may speculate that for these 4 participants, their available attentional capacity allowed them to easily maintain two conjunction templates, switching between them at little or no cost in speed or accuracy. As these individuals were able to reduce their overall movement by using the same strategy in the two conditions, we might consider them “super-foragers” in the same way that some “super-taskers” divide attention without apparent cost in the context of driving [38].

The behavior of one other participant also deserves particular mention. P13 stood out as having unusually long distance and response time measures in the feature condition (Figure 3). Close examination of the run pattern of this participant indicated that they adopted a very consistent strategy of regularly alternating between the two target categories, one after the other. This regular pattern explains the fact that they are the only participant with mostly non-random trial sequences in the feature condition (Table 1). It is unclear why P13 maintained this strategy as it was clearly very inefficient in terms of both time and distance travelled.

The behavior of these participants suggests that individual differences need to be taken into account when considering foraging patterns in humans, a call more generally being heard in the cognitive literature [39]–[40]. We additionally note that quite large individual differences in run patterns were noted in Bond's previous work, and that in a recent replication of the current experiment in our own lab, we again noted a similar proportion of “super-foragers” within a new group of participants. The implication of these findings, then, when attempting to define an “optimal” foraging strategy [33], [41] in a given environment, is that the cognitive capacity of the individual forager must be considered. If the cognitive-cost of switching internal templates – a possible analogue of the cost of moving between physical locations in the wild [23] – varies dramatically between individuals, this is likely to lead to very different foraging [27]. Determining whether such variability is uniquely human is a very exciting avenue for future research.

Finally, our current findings would seem to dovetail nicely with another series of studies that have recently examined human search performance in the context of foraging [36]–[37]. Building on previous serial-detection studies in other species [11]–[15], [35], human participants were asked to search for photographs of polymorphic variations of grasshoppers presented one-per-trial, on a variety of natural backgrounds. Detection performance was very sensitive both to the relationship between background and target coloration (i.e. extent of crypsis), and to the nature of the “runs” that were imposed on participants. Specifically, detection performance was enhanced when a single target type was presented several times in a row, in contrast to when target types were alternated. Similar switching costs have previously reported in blue jays [12]–[15] and are also consistent with repetition priming effects in studies of human search [30]. Together, these results suggest that the natural run behaviour we observed in our conjunction condition may have served to enhance individual target detection, as well as to avoid the cost of switching between categories. Although these studies of polymorphic variation did not directly measure foraging sequences, as we have done here, they clearly demonstrate that forcing a predator to “divide attention” is likely to influence search behaviour, and thus have potentially important consequences for prey survival, see also [35]. As we noted earlier, it would be very interesting to replace our current geometric stimuli with photographic targets and backgrounds to examine patterns of human foraging using more naturalistic stimuli.

## Conclusion

We have introduced a new task for studying foraging-like search behavior in humans. Our results clearly demonstrate that attention modulates the way humans search for targets across multiple categories, and more particularly, that it does so in a similar way to other species. These findings add to a growing body of research into human foraging that have replicated other key aspects of animal behavior, such as area-restricted search”[42], polymorphic search efficiency [37], Lèvy flights [24] and the predictions of Marginal Value Theorem [27]. Together with the current work, these findings add weight to claims for a common evolutionary thread that connects search behavior in animals to goal-directed cognition in humans [43]–[44].

## Supporting Information

File S1 **This file contains raw data from each of the 16 participants that took part in the current experiment.** The file is in CSV format and does not contain header information. The column labels (with explanations in parentheses) are as follows: 1) Participant Number; 2) Trial Number; 3) Experimental Condition (0 =  Feature, 1 =  Conjunction); 4) Symbol Condition (1 = R/G target, 2 = B/G, 3 = rSq/gDisk, 4 = gSq/rDisk); 5) Touch Count (1–40); 6) Target ID; 7) Target X position; 8) Target Y position; 9) Trial Time; 10) Touch Time; 11) Target Repetition (0 = no repeat. 1 = repeated); 12) Repetition Count; 13) Distance from last touch; 14) Run length; 15) Line number.
(CSV)
